# Crosstalk between Notch, HIF-1α and GPER in Breast Cancer EMT

**DOI:** 10.3390/ijms19072011

**Published:** 2018-07-10

**Authors:** Ernestina M. De Francesco, Marcello Maggiolini, Anna Maria Musti

**Affiliations:** 1 Department of Pharmacy, Health and Nutritional Sciences, University of Calabria, 87036 Rende, Italy; ernestina.defrancesco@manchester.ac.uk; 2 Breast Cancer Now Research Unit, Division of Cancer Sciences, Manchester Cancer Research Centre, University of Manchester, Wilmslow Road, Manchester M20 4GJ, UK

**Keywords:** notch signaling, GPER, estrogen, hypoxia, breast cancer, EMT, cancer associated fibroblast

## Abstract

The Notch signaling pathway acts in both physiological and pathological conditions, including embryonic development and tumorigenesis. In cancer progression, diverse mechanisms are involved in Notch-mediated biological responses, including angiogenesis and epithelial-mesenchymal-transition (EMT). During EMT, the activation of cellular programs facilitated by transcriptional repressors results in epithelial cells losing their differentiated features, like cell–cell adhesion and apical–basal polarity, whereas they gain motility. As it concerns cancer epithelial cells, EMT may be consequent to the evolution of genetic/epigenetic instability, or triggered by factors that can act within the tumor microenvironment. Following a description of the Notch signaling pathway and its major regulatory nodes, we focus on studies that have given insights into the functional interaction between Notch signaling and either hypoxia or estrogen in breast cancer cells, with a particular focus on EMT. Furthermore, we describe the role of hypoxia signaling in breast cancer cells and discuss recent evidence regarding a functional interaction between HIF-1α and GPER in both breast cancer cells and cancer-associated fibroblasts (CAFs). On the basis of these studies, we propose that a functional network between HIF-1α, GPER and Notch may integrate tumor microenvironmental cues to induce robust EMT in cancer cells. Further investigations are required in order to better understand how hypoxia and estrogen signaling may converge on Notch-mediated EMT within the context of the stroma and tumor cells interaction. However, the data discussed here may anticipate the potential benefits of further pharmacological strategies targeting breast cancer progression.

## 1. Introduction

The Notch signaling is an evolutionary conserved cell–cell communication mechanism operating in many cell types and at various developmental stages to determine cell-fate of neighboring cells. During mammal development, Notch signaling controls various aspects of organogenesis, including neurogenesis, vasculogenesis, myogenesis, and hematopoiesis [1]. Besides, the Notch pathway is also implicated in the regulation of cell proliferation and cell death [2]. In mammals, four different Notch transmembrane receptors can be activated by membrane-tethered ligands. Ligand interaction initiates a proteolytic cleavage of Notch receptors and the subsequent release of the Notch intracellular domain (NICD), which in turn translocates to the nucleus and regulates context-specific patterns of gene expression. This fairly simple core pathway is made complex by a number of secondary proteins, which can be activated by concurrent signaling pathways and modulate various steps of the Notch core pathway, so contributing to signal diversity. As predicted from Notch capacity to operate in several tissues, anomalous deregulation of this pathway has been associated with several human diseases, including cancer. Alteration of the Notch signaling pathway is a common feature of different types of malignancies, including hematopoietic and solid tumors. Several studies indicate that in T cell acute lymphoblastic leukemias (T-All), the occurrence of activating mutations in Notch genes is the principal causative factor initiating tumorigenesis, mostly through deregulation of cell-cycle progression and inhibition of apoptosis [3]. Differently, in solid tumors, multiple mechanisms underlie pro-tumorigenic activity of the Notch pathway. These mechanisms include the altered expression of Notch receptors and ligands, ligand-independent activation of Notch, anomalous regulation of Notch degradation and endocytic trafficking, as well as epigenetic control of Notch-dependent transcription [4,5]. Accumulating evidence indicates that the Notch pathway is a common avenue for epithelial mesenchymal transition (EMT) triggered by several tumor microenvironmental cues, including hypoxia and estrogens. In this regard, the alternate estrogen receptor GPER has been shown to induce EMT through activation of the Notch pathway [6]. Similarly, in different types of cancer cells hypoxia-activated HIF-1α has been shown to promote EMT by potentiating the Notch signaling pathway [7]. The aim of this review is to outline the most significant findings supporting the proposal that a signaling network between HIF-1α, GPER and Notch may integrate tumor microenvironmental cues to induce robust EMT in breast cancer cells. In the first part of this review, we describe the Notch core pathway, focusing on possible mechanisms regulating Notch endocytic trafficking and Notch target-gene selection in cancer cells. Following a briefly description of the current knowledge concerning the role of Notch signaling in cancer angiogenesis and EMT, we then discuss the most significant studies providing evidence for a functional crosstalk between estrogen and Notch signaling in breast cancer cells. Likewise, we highlight studies giving insights into the interplay between HIF-1α and Notch signaling in cancer EMT. In the second part of this review, we describe the role of hypoxia signaling in breast cancer cells and discuss recent evidence regarding a functional interaction between HIF-1α and GPER in both breast cancer cells and cancer-associated fibroblasts (CAFs). On the basis of these studies we propose that a signaling network between HIF-1α, GPER, and Notch, initiated by either HIF-1α in hypoxic cancer cell or E2/GPER in normoxic cancer cells, may account for Notch-dependent induction of Snail, a key transcriptional repressor of E-cadherin in breast cancer EMT.

## 2. The Notch Core Pathway

**Figure 1:**
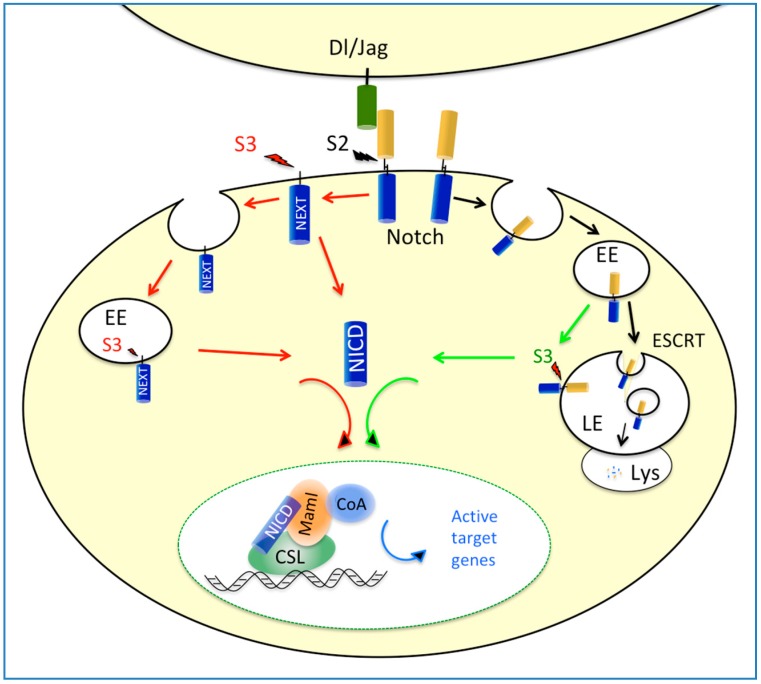
The Notch core pathway and endocytic routes to ligand-dependent and ligand-independent activation of Notch signaling. Red arrows indicate canonical paths of Notch activation initiated by Notch/Ligand (Dl/Jag) binding and generating Notch intracellular domain (NICD) molecules upon sequential proteolytic cleavage of Notch at site 2 (S2) and 3 (S3), by Adam metalloproteases and γ-secretase, respectively. Black arrows indicate the endocytic route of unbound Notch receptor regulated by the endosomal sorting complex required for transport (ESCRT) system and culminating with Notch degradation in late endosome (LE) fused to lysosome (Lys). Green arrows indicate the alternative endocytic path of unbound Notch, allowing Notch to escape degradation and whereas generation of NICD. Both red and green paths culminate with nuclear translocation of NICD and its recruitment at CSL-bound promoters, which triggers the exchange of CSL-bound co-repressor with Maml and co-activators of transcription.

Notch consists of a family of singlepass transmembrane receptors (Notch1–4), which can be activated by the interaction with membrane-tethered ligands, including Delta-like (Dll) 1–4 and Jagged (Jag) 1–2 [8]. The Notch native polypeptide consists of an extracellular domain (NECD) and a transmembrane intracellular domain (NTMIC). The newly synthetized Notch polypeptide is glycosylated throughout the NECD domain in the trans Golgi. The NECD is then cleaved at site 1 (S1) by a furin-like convertase to generate the NECD/NTMIC heterodimer receptor expressed on the cell surface. Upon ligand activation, a conformational change exposes NTMIC for a cleavage by ADAM metalloproteinases at site 2 (S2) generating the membrane-anchored Notch extracellular truncation (NEXT) fragment. In turn, NEXT is cleaved by γ-secretase at site 3 (S3) and releases the Notch intracellular domain (NICD). Thereafter, NICD translocates to the nucleus and regulates transcription of target genes by forming a complex with DNA-bound RBP-jk, also referred to as CSL, hence triggering the recruitment of co-regulator of transcription Mastermind-like (MAML) family member [9]. In absence of NICD, CSL interacts with various transcriptional repressors, which are then substituted by MAML in response to Notch activation (Figure 1).

This initial model of Notch-mediated transcriptional activation has been further characterized by several studies showing that CSL-binding co-repressors are mostly complexes of histone modifying enzymes keeping the chromatin in a compact inactive state; whereas MAML proteins recruit transcriptional co-activators, mostly p300/CBP, with histone-acetylation enzymatic activity [10]. Moreover, accumulating evidence suggests that the recruitment of NICD-CSL at promoter/enhancer induces dynamic histone epigenetic modifications [11], in turn favoring accessibility of either the tripartite complex NICD-CSL-MAML or repressor-bound CSL [12]. Such a mechanism could set a threshold concentration of the tripartite NICD-CSL-MAML complex for the displacement of the repressor-CSL complex from the target promoter; on the other side, it could guarantee a rapid switch off of Notch-dependent transcription upon decay of the inducing signal. Further studies are required to assess whether such a mechanism is context-specific or applies to most Notch-responsive genes.

## 3. Notch Endosomal Trafficking and Ligand-Independent Activation

A variety of post-translational events, including ubiquitination and endocytic trafficking have been shown to regulate the Notch core pathway, allowing context specific regulation. Upon ligand-dependent generation of the membrane-bound NEXT fragment, Notch activation proceeds through the γ-secretase-dependent proteolytic cleavage at S3 to release NICD, which mostly occurs at the cell surface. However, studies in Drosophila development have revealed that S3 cleavage of NEXT also occurs after internalization in early endosomes [13] and endosomal entry is required for efficient activation of the pathway [13] (Figure 1). In line, positive regulators of endosomal trafficking, such as Dynimin and Rab5, have been shown to promote canonical ligand-dependent activation of Notch signaling [14]. Furthermore, earlier studies in human cells have shown that Notch monoubiquitination is required for both the internalization in early endosome and γ-secretase cleavage [15]. Several studies suggest that the E3 ubiquitin ligase Deltex is the enzyme responsible for NEXT monoubiquitination and internalization in early endosomes [16]. Adding a further level to the regulation of Notch endocytic trafficking, Moretti et al. [17] showed that NEXT deubiquitination is also required for γ-secretase cleavage and release of NICD. Major negative regulators of Notch endocytic activation are Numb and Numb-like proteins, which alter the endocytic route of Notch receptor by promoting its degradation [18]. Besides their function in cell fate decision during development [18], Numb proteins act as tumor-suppressor of oncogenic Notch signaling during tumorigenesis [19].

In absence of ligand binding the Notch heterodimer undergoes endocytic turnover, a process requiring Notch polyubiquitination by the Su (dx)/Itch/AIP4 E3 ubiquitin ligase [20,21], in turn allowing Notch internalization in early/late endosomes. In late endosomes, multiubiquinated Notch is then sorted to the multi vesicular body (MVB), through a serial-steps mechanism operated by the endosomal sorting complex required for transport (ESCRT) system; finally, upon MVB fusion with lysosomes, the Notch receptor is eventually degraded [22] (Figure 1). Studies in Drosophila mutants have shown that deficiencies of various components of the ESCRT system lead to ligand-independent activation of Notch, through a mechanism allowing γ-secretase cleavage in late endosomes and the consequent release of NICD (Figure 1) [22,23,24].

Moreover, studies in Drosophila crystal cells indicate that endocytic activation of unligated Notch is regulated by Sima, the ortholog of mammal HIF-1α [25]. This study shows that Sima promotes crystal cell survival by allowing accumulation of full-length Notch in early endosome and its subsequent ligand-independent activation. Furthermore, this study reports that overexpression of Rab5 suppresses the effect of Sima on crystal cell survival, presumably by increasing Notch endocytic turnover. Notably, HIF-1α was previously demonstrated to downregulate the Rab5 effector rapabtin-5, thereby the endocytic pathway [26]. These results suggest that the regulation of Notch endocytic sorting may represent a further point of crosstalk between Notch and signaling pathways activated by tumor microenvironmental cues. It would be interesting to assess whether in human cancer cells, hypoxia-activated HIF-1α may lead to ligand-independent activation of Notch signaling by mimicking the effect of Sima on Notch endocytic trafficking. Thus far, there is no direct evidence for oncogenic mutations targeting the Notch endocytic pathway in human cancer. However, Notch endocytic sorting and proteolytic maturation has been shown to occur also in mouse and human cells [27]. In line, vacuolar H^+^ATPase (V-ATPase), whose activity is required for acidification of endocytic organelles, was shown to be required for endosomal cleavage of NEXT by γ-secretase, both in Drosophila and human cells [28]. In particular, this study has shown that in normal human breast cells, pharmacological inhibition of either V-ATPase (by BafA1) or γ-secretase (by DAPT) reduced with similar efficiency the steady-state levels of NICD, as well as the expression levels of the Notch target gene *Hes1*. These results indicate that endocytic maturation of Notch is the major route for Notch activation in normal breast cells. Yet, the authors showed that in the breast cancer cell line HHCC1599, harboring a gene rearrangement of Notch1 causing ligand-independent, but γ-secretase dependent activation of Notch [29], BafA1 had a smaller effect than DAPT in reducing the steady-state levels of NICD and Hes1. This observation suggests that constitutive expression of oncogenic Notch, structurally similar to the NEXT fragment, favors γ-secretase cleavage at the plasma membrane. Interestingly, previous studies by Tagami and collaborators [27] indicated that depending on the subcellular location of NEXT proteolytic cleavage, the precise location of the S3 cleavage-site by γ-secretase can shift to the next few residues, producing NICDs that vary in their PEST domain and consequently in their stability. Moreover, these various form of NICDs generated different intensity of Notch signaling [27]. In particular, this study showed that the NICD generated at the plasma membrane was the major form present in Hela cells and was more stable than the NICD generated in early endosome. These observations indicate that the specific subcellular location of NEXT proteolysis may determine the duration of Notch signaling in signal-receiving target cell. Taken together, these studies suggest that certain oncogenic variations of Notch protein structure may strengthen Notch signaling by altering the subcellular location of γ-secretase proteolysis, in turn enhancing Notch signaling in cancer cells.

## 4. NICD Transcriptional Strength and Target Selection

Depending on spatial and temporal context, the functional output of Notch signaling may be radically different, ranging from cell stemness to differentiation and cell death. As described above, Notch-dependent transcription is based mostly on the CSL–NICD–MAML transcriptional complex, yet capable of inducing context-specific gene expression patterns. A recent review discussed the wide number of studies providing important insights into the possible factors governing context-specific expression of Notch target genes [8]. Here, we will briefly consider those factors that may influence target-gene selection by modulating NICD stability, or by regulating the cooperation of NICD with specific transcription factors.

Since Notch acts as a ligand-activated transcription factor, the expression levels of both membrane-bound Notch and the processed intracellular NICD are crucial for the signaling strength and duration of the signaling, as proved by Notch1 haploinsufficiency in several diseases [30]. NICD stability is mostly controlled by the E3 ubiquitin ligase Fbw7, which targets the NICD C-terminal domain for ubiquitination and consequent degradation [31]. Several studies have shown that Notch oncogenic mutations map in the Fbw7 degron domain of Notch, avowing NICD degradation and leading to reinforcement of Notch signaling [10]. Similarly, in vivo abrogation of Fbw7 expression in mesenchymal stromal cells (MSCs) induces NICD stabilization and the consequent up-regulation of the CCl2 chemokine, which in turns promoted breast cancer metastasis in mice [32]. CCl2 expression in MSCs was shown to be strictly dependent on NICD recruitment at the promoter of *CCL2* gene, suggesting that environmental variation of Fbw7 expression might determine the selection of novel Notch-target genes in the tumor microenvironment. Interestingly, Fbw7 expression is negatively regulated by extrinsic cues activating oncogenic signaling [33,34].

Certainly, the context-specific expression pattern of canonical ligands and receptors also participate to the diversity of Notch functional output and represent an important crosstalk road with other signaling pathways [8]. However, studies from Liu at al. [35] suggest that the different biological outputs of Notch-1 and Notch-2 may reflect different strengths of the respective signals. In particular, this study shows that the structural differences present in the NEXT fragments generated by Nocht-1 or Notch-2 receptors affect the subcellular location of their respective S3 cleavage by γ-secretase, with the Notch2-NEXT being more frequently cleaved at the cell surface than the Notch1-NEXT. Interestingly, the NICD/Notch2 resulted in having greater signal strength than the NICD/Notch1, confirming previous studies by Tagami et al. (discussed above) showing that the subcellular location of NEXT proteolytic cleavage can determine the strength of Notch signaling [27]. Together these studies suggest that context-dependent location of S3 cleavage of NEXT fragments may contribute to gene-target selection by discriminating between genes responding to different transcriptional strength of the Notch signaling.

Genome wide studies have indicated that NICD/CSL complex occupies only a limited number of the CSL canonical motif present in the genome [36]. This observation suggests that other transcription factors (TF) may promote the recruitment of NICD/CSL complex at specific promoters or enhancers, so contributing to gene-target selection. For instance, studies in T-lymphoblastic leukemia cells have shown that CSL binding motifs are often located in enhancers containing histone modifications typical of active chromatin, which favor DNA accessibility [37]. This study also shows that within several of these active enhancers, the CSL binding site overlaps with that of Runx, a TF required for T-cell development [37]. Notably, the study demonstrated the requirement of Runx for the expression of Notch-target genes, suggesting that cooperation of NICD/CSL with lineage specific TFs may be crucial for Notch-target selection. Cooperation with signal-induced TFs may also augment CSL-NICD activity at specific target genes. For example, a study by Sahlgren et al. (discussed later in this review) has shown that in human ovarian carcinoma cells hypoxia-activated HIF-1α is recruited together with NICD at the promoter of the Notch-target *Snail-1* gene, hence increasing *Snail-1* expression [38]. Similarly, β-catenin is recruited at the promoter of Notch-target genes during the differentiation of arterial endothelial cells from vascular progenitor cells [39].

## 5. Notch Signaling in Tumor Angiogenesis and EMT

### 5.1. Angiogenesis

Angiogenesis consists in the generation of new blood vessels from preexisting vasculature. In normal tissues, angiogenesis is initiated by hypoxia-stimulated production of the vascular endothelial growth factor (VEGF), which stimulates the formation of a new sprout, whose very front cell is called a “tip” cell. In response to VEGF, the tip cell extends several filopodia towards the VEGF gradient, whereas the adjacent endothelial cells, named stalk cells, do not respond to VEGF, but proliferate and form the lumen of the branching vessel [40]. This selection of the tip and the stalk cell fate is critical for successful angiogenesis and is based on the type of Notch ligands expressed on the tip and stalk cells. In particular, the tip cell is stochastically-determined by VEGF stimulation, which in turn induces the expression of the Notch ligand Dll4. In turn, Dll4 induces Notch signaling in the adjacent endothelial cell expressing Notch receptors. Through an inhibitory mechanism, named lateral inhibition [8], Notch signaling inhibits the expression of Dll-type of receptors and VEGFR2, hence determining the peculiar stalk cell phenotype [40]. The VEGF/Dll4/Notch pathway also functions in cancer angiogenesis, where changes in the expression levels of signaling molecules, including Notch ligands Dll4 and Jag1, lead to altered morphological features of the vascular endothelium [41,42,43]. Furthermore, in tumor models, blockade of Dll4-Notch signaling results in vessel hypersprouting, nonfunctional tumor vasculature, and inhibition of tumor growth [44,45]. Besides, deregulated expression of Notch ligands in endothelial cancer cells may also regulate invasiveness by promoting Notch activation of adjacent cancer cells [46]. Similarly, Jag1 secreted from tumor endothelial cells promotes the cancer stem cell phenotype of colorectal carcinoma cells [47].

### 5.2. EMT

Epithelial-mesenchymal transition (EMT) is a conserved process occurring during both embryonic development cancer and progression, through which polarized epithelial cells become migratory mesenchymal stem cells [48]. During embryogenesis of both invertebrate and vertebrates, multiple rounds of EMT are crucial for the formation of tissues and organs [48,49]. In cancer progression, EMT confers migratory and invasive features to epithelial cancer cells and is mainly achieved through transcriptional repression of junctional proteins, among which the cell–cell adhesion molecule E-cadherin is repressed in several invasive carcinomas [48]. Various highly conserved signaling pathways operating in embryonic development are known to trigger EMT in cancer cells. Among these pathways, Wnt, TGF-beta and Notch signaling converge at the induction of direct transcriptional repressors of E-cadherin, including Snail/Slug, Twist and ZEB1/2 [48,50]. Accumulating evidence suggest that Notch-dependent up-regulation of Snail/Slug expression mediates the induction EMT by interacting signaling pathways, as TGF-beta, hypoxia, and estrogen/GPER [6,38,51,52]. Being downstream of multiple signaling pathways leading to EMT, Snail expression positively correlates with metastasis and poor prognosis in different types of tumors [50]. Snail has been shown to repress E-cadherin expression by recruiting histone-modifying enzymes at the *CDH1* gene promoter (coding E-cadherin protein), including Sin3A-HDAC1/2 [53], Polycomb complex 2 (PRC2) [54] or Lysisn-specific demethylase 1 (LSD1) [55], which turn chromatin into an inactive state. In different types of cancer cells, Notch signaling promotes EMT by directly inducing Snail expression [6,38,56,57]. At transcriptional level, Notch regulates Snail expression through the tripartite complex NICD-CSL-MAML-1, as demonstrated by the evidence that the dominant-negative mutant of MAML-1 (DN-MAML-1) inhibits Snail expression and EMT in cancer cells [6,38]. As discussed later in this review, Notch also controls Snail protein expression by an indirect mechanism augmenting Snail protein stability in hypoxic cancer cells [38].

## 6. Interplay between Notch Signaling and 17β-Estradiol (E2) in Breast Cancer

Earlier studies in breast cancer reported that high expression levels of Notch1 and Jag1 were associated with poor prognosis [58], suggesting a possible interplay between estrogen and the Notch signaling pathway. Furthermore, Stylianou et al. [59] observed accumulation of NICD in a wide variety of human breast cancer cell lines and tissue samples, indicating that the Notch pathway may be aberrantly activated in human breast cancer. Estrogens mainly act through the cognate receptors namely ERα and ERβ, which upon hormone binding translocate to the nucleus and function as transcription factors regulating estrogen-responsive genes [60]. Studies by Rizzo et al. [61] documented a negative crosstalk between ERα and the Notch signaling in breast cancer cells. Results from this study suggest that E2-activated ERα causes an accumulation of inactive membrane-bound Notch1, which was in part due to inefficient γ-secretase cleavage of the Notch receptor. However, E2 did not cause inhibition of γ-secretase activity, suggesting that E2-activated ERα may effect Notch endocytic trafficking. A negative interplay between Notch1 and ERα was confirmed by Haughian et al. [62], as in ER^+^ luminal breast cancer Notch-1 signaling mediated the expansion of a luminobasal ER^−^ subpopulation of cells, which arose upon endocrine therapy. Interestingly, Stinson et al. [63] identified miR-221/222 as the two miRNAs whose expression pattern can clearly discriminate between the ER^−^ luminobasal and the ER^+^ luminal subtypes of breast cancer. Moreover, the transfection of miR-221/222 transfection in a human mammary epithelial cell line promoted EMT by targeting TRPS1, a member of the GATA family transcriptional repressor [64], which repressed the expression of the EMT transcription factor ZEB2. Interestingly, a recent study by Golan et al. [65] showed that Notch-1 activation in melanoma cells directly derepresses miR-221/222 expression, therefore promoting melanoma invasion. Notably, the *ESR1* gene (encoding ERα) resulted in a major target of miR-221/222 and its knockdown mirrored the effect of miR-222 overexpression on melanoma invasive activity. Furthermore, Han et al. reported that in ER^+^ breast cancer, miR-222 is associated with down-regulation of ERα, EMT, and tumor progression [66]. On the other hand, earlier studies in breast cancer showed that ERα promoted metastasis-associated family member 3 (MTA3)–dependent repression of Snail and E-cadherin expression [67]. A few years later, the same group proposed an inverse mechanism, showing that in non-invasive MCF7 cancer cells, Snail induces EMT features by directly repressing ERα expression [68]. A recent study reported a similar mechanism of ERα repression by Slug in human breast cancer [69]. Taken together, these studies suggest a mutual functional repression between Notch1 and ERα signaling in breast cancer EMT, where ERα-responsive genes (e.g., *MTA3*) would repress the expression of EMT-transcription factors laying downstream the Notch pathway. Conversely, Notch target genes (e.g., miR-221/222 or Snail) would repress ERα expression. Presumably, in normal breast epithelial cells these two opposing signaling pathways act in an opposite manner, whereas during malignant transformation, either intrinsic variations or microenvironmental signals may disrupt this balance and favor the expression of aggressive phenotypes in breast cancer.

Beyond ERα and ERβ, estrogens signal through the G-protein coupled estrogen receptor, a member belonging to the rhodopsine-like family of G-protein coupled receptors (GPCRs) [70]. GPER has been shown to unlock access to estrogens in breast cancer cells devoid of the classic Estrogen Receptor (ER), thus allowing the stimulatory actions of these steroids [70]. In estrogen-responsive tumors, GPER expression correlates with negative clinical-pathological features such as larger tumor size, distant metastasis, and worse prognosis [71,72,73,74]. In accordance with these observations, GPER signaling has been linked to ERα loss, which occurs in breast cancer cells undergoing tamoxifen resistance [75]. GPER mediates rapid estrogen action via heterotrimeric G proteins, hence activating multiple intracellular pathways regulating crucial mechanisms involved in breast cancer growth, invasion, and metastasis [76]. As discussed in the next sections, GPER also signals in cancer-associated fibroblasts (CAFs), therefore contributing to the expression of pro-tumorigenic factors within the tumor microenvironment, as inflammatory cytokines and angiogenic factors [77]. Regarding breast cancer invasiveness, previous studies showed that in ER-negative breast cancer cells or CAFs, E2/GPER signaling induced cell migration through EGFR-dependent activation of connective tissue growth factor (CTGF) [78,79]. The following studies showed that GPER interacts with multiple signaling pathways culminating with the expression of various molecules inducing cell migration in breast cancer cells [80,81,82]. As it concerns Notch signaling, our previous studies showed that estrogen engages a positive crosstalk between GPER and the Notch pathway, triggering Notch/Snail-mediated EMT in both ERα positive and ERα negative breast cancer cells, as well as in CAFs [6]. In particular, we demonstrated that E2/GPER signaling induced ligand-independent activation of Notch1 and Notch target genes, including the EMT-transcription factor Snail, whose activation required the tripartite NICD-CSL-MALM-1 transcriptional complex. Most importantly, breast cancer cells acquired invasion properties and lost Cadherin expression in a GPER/Notch dependent fashion. Of note, our study showed that E2/GPER signaling induced both Notch1 mRNA and NICD protein levels [6]. Further studies are required to elucidate whether and how the increase of NICD levels observed by GPER signaling is directly linked with ligand-independent activation of Notch.

## 7. Crosstalk between HIF-1α and Notch in Cancer EMT

As discussed in the next Section, the homeostatic function of hypoxia during normal development and in pathophysiology is mostly mediated by the activation of HIF-1α transcription factor and the subsequent induction of its target genes, primarily acting in angiogenesis [83] and energy metabolism [84]. Hypoxia-activated HIF-1α is also involved in cellular processes controlling phenotypes of stem cells during embryogenesis [85], as well as in cancer stem cells (CSCs) during tumor initiation and progression [86]. Likewise, the Notch signaling pathway has been shown to be crucial for stem cell maintenance and cell fate control, both during development and cancer progression [87]. Such a functional similarity between these two signaling pathways has prompt researchers to assess possible functional interactions, both in developmental and cancer biology. Indeed, as remarkably discussed in a recent review [7], several studies have shown that certain functional outputs of the hypoxic response, as the regulation of stem cell differentiation [88], require the expression of Notch target genes. As regards cancer, first hints for a HIF-1α/Notch crosstalk came from a transcriptomic analysis showing the upregulation of Notch target genes in hypoxic neuroblastoma cells [89]. Recently, a gene expression analysis of Notch and hypoxia-activated genes in glioblastoma tumors confirmed a combined gene signature of these two pathways and its relevance in tumor prognosis [90]. Same trends have been reported in different types of brain tumors [7].

EMT confers migratory and invasive features to epithelial cancer cells and is mainly achieved through transcriptional repression of E-cadherin. As discussed earlier, Notch signaling represses E-cadherin expression mostly through the induction of Snail/Slug transcriptional effectors [48]. Hypoxia is often an environmental feature of the tumor invasive front, where the EMT program takes place. Hypoxia-activated HIF-1α induces cancer EMT through diverse molecules and pathways, including inflammatory cytokines, epigenetic regulators, and transcription factors/repressors [91]. As it concerns the induction of Snail by hypoxia, first indications came from studies in ovarian cells lines, where HIF-1α was shown to induce EMT through up-regulation of Snail expression and consequent repression of E-cadherin [92]. Snail expression is regulated at multiple levels, including transcriptional expression and protein stabilization [50]. Of note, hypoxia has been shown to influence both levels of Snail regulation. For instance, hypoxia-induced HIF-1α was shown to indirectly inhibit Snail ubiquitination by inducing Sox-9–dependent expression of the USP47 deubiquitinase in lung cancer cells [93]. On the other hand, hypoxia was shown to induce Snail regulation through a synergistic mechanism between Notch-NICD and HIF-1α [38]. Firstly, hypoxia indirectly induced Snail-1 transcription by promoting a HIF-1α/NICD physical interaction, which in turn increased the recruitment of NICD to the Snail-1 promoter; next, Notch increased the recruitment of HIF-1α to the lysyl oxidase (LOX) promoter region, hence triggering LOX expression. As a consequence, LOX stabilized Snail protein levels, which migration and invasion of breast cancer cells [38]. A further study confirmed the role of Notch signaling in mediating the effect of hypoxia toward the activation of Snail and Slug in breast cancer cells [94]. In particular, this study showed that HIF-1α binds to the promoter of Notch target genes Hes1 and His2 and cooperates with the transcriptional coactivator MALM1 to potentiate transcriptional events. Similarly, HIF-1α was shown to induce EMT in oral squamous cell carcinoma by inducing Notch-dependent activation of Snail [95]. As mentioned above, Snail represses E-cadherin expression by recruiting different histone-modifying enzymes at the CHD1 promoter [96]. Regarding this topic, HIF-1α was shown to induce the expression of HADC3, which cooperated with Snail to repress E-cadherin expression along with other epithelial-specific genes [97]. It remains to be elucidated whether such a mechanism also contributes to the repression of E-cadherin mediated by Notch signaling in hypoxic cancer cells.

On the other hand, Villa and collaborators discovered that hypoxia-activated HIF-1α induces Notch signaling independently from its transcriptional activity [98]. The mechanism consists of a direct interaction between HIF-1α and γ-secretase that enhances γ-secretase activity and Notch cleavage at site S3. In line with this model, the aforementioned study showed that inhibition of either γ-secretase activity or a transcriptional coactivator of Notch-NICD prevents the invasiveness of breast cancer cells [98]. The non-transcriptional role of HIF-1α in the activation of Notch signaling was further confirmed in lung adenocarcinoma cancer cells, where cadmium exposure was shown to induce EMT by Notch-dependent expression of Snail, in a HIF-1α dependent manner [57].

## 8. Hypoxia-Inducible HIF-1α in Breast Cancer Cells and CAFs

### 8.1. HIF-1α in Breast Cancer Cells

Solid malignancies like breast cancer possess a unique microenvironment often characterized by hypoxia [99,100]. Low oxygen (O_2_) occurs in certain neoplastic areas when the balance between O_2_ consumption and O_2_ supply is lost. Indeed, the highly metabolic demands of cancer cells, the disorganized and leaky tumor vasculature, as well as the mechanic pressure exerted by the growing tumor mass on existing capillaries and blood vessels, all together limit O_2_ diffusion and reduce its availability [99]. Hypoxia, which has been included among the hallmarks of cancer, is associated with genetic instability and chemo/radio-resistance, as well as disease progression and evolution toward metastatic phenotypes [100,101]. Thereafter the broad range of molecular and cellular responses triggered by low O_2_ tension in the tumor microenvironment is currently under the magnifying lens for the enormous clinical potential [102,103]. In breast cancer, regions of hypoxia have been detected in all stages, however only tumors with worse prognostic features, as lack of estrogen receptor (ER) and progesterone receptor (PR) or node-positive status, are characterized by higher degree of hypoxia and major extent of low O_2_ areas [104]. The pivotal molecular mechanisms and biological responses influencing breast cancer outcome in hypoxic microenvironment are triggered by the hypoxia inducible factors (HIFs) [105,106,107]. HIF-1, HIF-2, and HIF-3 are oxygen-regulated, basic helix-loop-helix (bHLH)-containing PER-ARNT-SIM (PAS) transcription factors which sense and respond to fluctuation of O_2_ levels [108,109,110]. While HIF-1 is expressed ubiquitously in human cells, the expression of HIF-2 and HIF-3 is restricted to certain tissues [111]. Furthermore, it has been established that HIF-1 acts as an immediate but transient effector in severe hypoxia, while HIF-2 responds to moderate drop in O_2_ levels [112]. HIF-1α has long been recognized as a pivotal orchestrator of the cancer cell responses to hypoxic microenvironment, by regulating the expression of genes involved in metabolic reprogramming and pH balance, cell proliferation/survival, apoptosis, angiogenesis, stem cell maintenance, matrix remodeling, metastasis, and resistance to radio- and chemo-therapy [107]. Like all the other members of the HIF family, HIF-1 is composed of both an O_2_-dependent alpha subunit and an obligate binding-partner, the aryl hydrocarbon nuclear translocator (ARNT), also known as HIF1-β [109,110]. HIF-1α expression depends on O_2_ levels, being almost undetectable in normoxic conditions, whereas HIF-1β is constitutively expressed. Under aerobic conditions, HIF-1α levels are maintained very low due to a tightly regulated mechanism that requires the participation of Prolyl Hydroxylase Domain (PHD) enzymes and the von Hippen Lindau (VHL) tumor-suppressor protein, as well as the engagement of the proteasome [113]. In the presence of O_2_, PHD enzymes hydroxylate HIF-1α subunit at specific proline residues, including Pro402 and Pro564; this reaction requires not only O_2_, but also iron (Fe^2+^) and alpha-ketoglutarate (αKG) as co-factors. Hydroxylation of HIF-1α at proline residues serves as a label for HIF-1α binding to the VHL-E3 ubiquitin ligase complex (composed of elongin B, elongin C, cullin 2, andring-box 1). As a consequence, HIF-1α is ubiquitinated and then degraded by the proteasome. Beyond PHD enzymes, HIF-1α activity is negatively regulated by the asparaginyl hydroxylase named FIH-1 (factor-inhibiting HIF-1), which hydroxylates HIF-1α at a conserved asparagine residue (Asn-803), thus preventing the binding to the transcriptional coactivator CBP/p300 which is required for transcriptional activity [114,115].

Under hypoxic conditions, the prolyl and asparaginyl hydroxylation of HIF-1α is inhibited, resulting in accumulation of HIF-1α/HIF-1β dimers, hence recruitment of transcriptional co-activators to the promoter of specific target genes carrying the hypoxia-response elements motif, identified as the core sequence 5′-(A/G) CGTG-3′ [116,117]. As pivotal players of HIF-1α regulation, PHDs and VHL are frequently deregulated in breast cancer and may serve as useful prognostic markers. In this regard, positive staining for hydroxylated HIF-1α at both VHL binding sites (Pro402 and Pro564) identifies a subset of breast cancer patients with poorer prognosis [118]. Similarly, analysis from primary breast cancer samples and human breast cancer cell lines revealed that low levels of VHL correlate with invasive and migratory capacity, as well as with aggressive features of breast disease [119]. Moreover, results from a randomized trial enrolling 211 breast cancer patients undergoing neoadjuvant epirubicin therapy showed upregulated expression of PHD1, PHD2 and PHD3, together with HIF-1α, VEGF and carbonic anhydrase IX (CAIX) [120]. Taken together these studies support the involvement of PHDs and HIF pathways in response to chemotherapy and possibly involved in chemo-resistance. Interestingly, a new physiologically relevant methylation-based mechanism was shown to regulate HIF-1α stability. In particular, methylation of HIF-1α on the 32nd lysine residue by the nuclear SET7/9 methyltransferase triggers HIF-1α degradation in normoxia, whereas the demethylase LSD1 removes the methyl group, thus increasing HIF-1α protein stability upon hypoxic conditions [121]. Importantly, analysis on knock-in mice expressing a methylation-resistant HIF-1α mutant protein confirmed that lysine demethylation is a crucial mechanism regulating hypoxia-induced stability of HIF-1α in breast cancer [121].

### 8.2. HIF-1α in CAFs

HIF-1α expression is tightly regulated also within the breast tumor microenvironment, which is recognized as a critical player for disease development and progression [122]. Indeed, endothelial cells, macrophages, adipocytes, and activated fibroblasts in breast cancer contribute to relevant biological responses, including cell proliferation, invasion, metastasis, angiogenesis, immunosuppression, and therapeutic resistance, ultimately leading to the acquisition of malignant features of breast disease [123,124,125]. Cancer-associated fibroblasts (CAFs) represent the most abundant cell type in breast cancer stroma and mostly derive from normal stromal fibroblasts (NFs) [126,127]. Only a small subpopulation of fibroblasts is quiescent within the breast tumor mass, whereas the majority of fibroblasts show an activated phenotype characterized by the ability to produce various ECM components and paracrine mediators, including growth factors and cytokines [128]. Among the several mechanisms involved in the transition of NFs towards CAFs, local hypoxia was shown to drive the differentiation of NFs into activated myofibroblasts by triggering the formation of Reactive Oxygen Species (ROS), which generate an elevated amount of hydrogen peroxide themselves, thereby boosting the biological responses to hypoxic stress [129,130,131]. In accordance with these observations, ROS-triggered and HIF-1-dependent metabolic and transcriptional reprogramming in CAFs was shown to promote EMT and metastasis [132]. Furthermore, it was shown that loss of PHD2 (EGLN1) impairs tumor growth and suppresses the pro-metastatic activity of CAFs [133,134]. In addition, hypoxia-dependent depletion of PHD2 deactivates CAFs through the loss of αSMA, periostin, and myosin II. In line with these findings, the PHD inhibitor DMOG was shown to significantly decrease spontaneous lung and liver metastasis in an orthotopic breast cancer model [135]. Likewise, in a spontaneously arising PyMT-oncogene driven breast cancer model (MMTV-PyMT model), loss of PHD2 is associated with vessel normalization, a reduced CAF activation and a decreased metastatic intravasation [134]. Altogether, these findings suggest that the blockade of PHD2 in CAFs may represent a novel strategy to suppress pro-metastatic signals within the breast tumor microenvironment.

Extending these findings, CAFs action through HIF has been shown to include the regulation of metabolic inter-dependencies between cancer cells and the surrounding microenvironment. Indeed, HIF-1α is involved in the regulation of a two-compartment metabolic symbiosis between anabolic cancer cells and catabolic stromal fibroblasts [136]. In particular, the increased glycolytic rates exhibited by CAFs are at least in part dependent on HIF signaling [136]. In addition, the high-energy metabolic by-products produced by catabolic CAFs are taken up by tumor cells to support their high anabolic requirements [137]. Interestingly, the elevated metabolic flux of cancer cells generates ROS, which are then propagated within the tumor microenvironment and in CAFs to promote HIF-dependent metabolic reprogramming [138,139].

### 8.3. Cytokines Regulating HIF-1α and NOTCH Signaling in the Tumor Microenvironment

In the tumor microenvironment, the balance between pro- and anti-inflammatory cytokines deeply influences breast cancer biology, providing a causal connection between inflammation and disease progression. Autocrine and paracrine actions elicited by cytokines and chemokines in the tumor microenvironment control the intricate communications occurring between cancer cells and stromal components toward the acquisition of malignant features [140]. Not surprisingly, several cytokines activate the HIF-1α pathway, actually evoking a hypoxic-like response in the tumor mass. In addition, many of the tumor-promoting effects induced by cancer-derived cytokines involve Notch-mediated action as the main trigger. Supporting these observations, pro-inflammatory cytokines are known to induce HIF-1α expression in normoxia [141,142], nevertheless cytokine-dependent effects are also observed in the hypoxic inflammatory tumor microenvironment [143]. In this context, tumor stromal components play a relevant role in mediating paracrine actions occurring at the interface between cancer cells and the hypoxic and inflammatory microenvironment. For instance, the cytokine Oncostatin M, belonging to the Interleukin-6 (IL-6) family of cytokines, enhances HIF-1α protein stability and induces HIF-1α de novo synthesis via activation of mTORC2 in breast Tumor Associated macrophages (TAMs), leading to inflammatory reprograming [144]. Worthy, CAFs isolated from breast tumor carcinomas promote angiogenesis by recruiting endothelial progenitor cells through stromal cell-derived factor 1 (SDF-1)/CXCR4 pathway [145], which is known to be activated by HIF-1α in several stressful conditions [146]. Likewise, cytokines as TGF-β and IL-1β may be released by breast cancer cells in a HIF-1α dependent manner and promote the conversion of normal fibroblasts to CAFs, hence encouraging tumor growth, metastatic spread, and neoangiogenesis [147]. Interestingly, in breast cancer cells both HIF-1α and TGF-β regulate a common set of genes including VEGF and CXCR4, toward the activation of the metastatic program, whereas the combined use of small molecule inhibitors of HIF-1α and TGF-β, targeting cancer cells and the microenvironment, reduces bone metastasis by decreasing osteoclastic bone resorption and increasing osteoblast activity [148]. Extending these findings, the HIF-1α-mediated release of TGF-β in hypoxic breast tumor microenvironment triggers Mesenchymal Stem Cells (MSCs) activation, thereby promoting growth, motility and invasive effects in breast cancer cells [149]. Of note, the tumor suppressor gene *TAp73* elicits anti-cancer effects by simultaneously blocking HIF-1α and the pro-inflammatory chemokines Ccl2, Cxcl1, Cxcl2, which are known to promote angiogenesis and leukocyte migration [150]. These observations support the idea of shared regulatory pathways that control both HIF-1α and cytokine signaling in the tumor microenvironment, orchestrating paracrine interactions that facilitate tumor progression.

It’s worth mentioning that cytokines produced within the breast tumor stroma promote cancer cell growth also by engaging the HIF-1α/Notch signaling. For instance, using mammosphere assay as a readout for CSCs activity, Sanguinetti and coworkers found that IL-6 induces stem-like features by stimulating the Notch-3-dependent upregulation of the carbonic anhydrase IX gene and promoting a hypoxia-resistant/invasive phenotype in breast cancer cells [151]. In addition, IL-6 derived from metastatic CAFs triggered breast cancer growth in vitro and in vivo through the involvement of Notch-3, Jagged-1, and the HIF-1α target gene carbonic anhydrase IX [152].

On the other hand, in the hypoxic breast tumor microenvironment, HIF-1α triggers Notch-3 up-regulation toward the decrease of IL-6 levels and the inhibition of the CSCs population, whereas the gamma secretase inhibitors MK-0752 and RO4929097 increase the CSCs population in an IL-6 dependent manner [153]. These observations suggest that coupling Notch inhibitors with cytokine-disrupting agents may serve as effective therapeutic strategy for breast cancer patients, although the contribution of several microenvironmental factors, including local hypoxia, should also be considered. Based on these observations, the cross-talk between HIF-1α and Notch signaling pathways may serve as a molecular bridge connecting together the multiple paracrine interactions occurring between breast cancer cells and CAFs toward disease progression.

## 9. Hypoxia-Independent Activation of HIF-1 in Cancer and Stromal Cells

Although both prolyl and asparaginyl hydroxylases represent canonical components of the O_2_-sensing cell machinery, HIF-1α activation in cancer may rely on additional non-canonical pathways, including several O_2_-independent molecular mechanisms. In this context, loss of VHL expression or function, as well as activation of chaperones like RACK1, were shown to play an integral role [154,155,156]. Although modulation of HIF-1α protein stability has been classically regarded as the main mechanism regulating HIF1α-dependent function, it is now evident that changes in HIF-1α mRNA expression levels and induction of protein synthesis can be accountable for certain molecular and biological actions mediated by this transcription factor, particularly in breast cancer. Several extrinsic and intrinsic cues, including oncogene activation, growth factors, hypoxia, cytokines, pH, and free radicals were shown to induce major pro-survival pathways, including the ERK/MAPK, JAK/STAT and PI3K/AKT/mTOR cascades, in turn regulating both HIF-1α gene expression and protein translation, hence promoting tumor progression, invasion and therapeutic resistance [157,158]. In this regard, it should be mentioned that aberrant Receptor Tyrosine Kinase (RTK) signaling, which is frequently observed in human cancer, is at least in part responsible for the increased up-regulation of HIF-1α mRNA and/or protein expression. For instance, EGFR and Her2 signaling have been shown to increase the rate of HIF-1α synthesis and the subsequent expression of survivin and VEGF in breast cancer cells, through the involvement of the PI3k/AKT pathway and the downstream kinase FRAP (FKBP-rapamycin-associated protein) [159]. This study suggests that EGFR- and HER2-mediated induction of HIF-1α expression may play a relevant role in breast tumor angiogenesis and progression. Of note, HIF-1α expression in normoxic conditions was shown to mediate certain detrimental effects dependent on HER2 action and involved in the resistance to aromatase inhibitors in breast cancer cells [159].

Several lines of evidence have suggested that the IGF-IR/IR signaling is implicated in the activation of the HIF-1α pathway in breast cancer. In ER-positive breast cancer cells, IGF-I was shown to induce HIF-1α protein translation through the PI-3K/AKT/mTOR-pathway and promote its transcriptional activity, without affecting HIF-1α mRNA levels [160,161]. On the other hand, in ER-negative breast cancer cells and CAFs, IGF-I up-regulated both HIF-1α mRNA and protein levels through the activation of AKT and MAPK transduction pathways [162].

Recently, the chemotherapeutic agents, paclitaxel or gemcitabine, were shown to induce HIF-1α transcriptional activation through elevation of ROS levels and in turn promote the chemo-resistance of breast cancer stem cells [163].

Elevation of ROS levels is crucial also for the copper-dependent induction of HIF-1α expression in breast cancer cells [164]. In this study, we showed that the ROS scavenger NAC abrogated the effect of copper on the activation of the EGFR/ERK/c-fos transduction pathway leading to the expression of HIF-1α, GPER and VEGF in breast and hepatic cancer cells. Furthermore, we demonstrated that a functional cooperation between HIF-1α and GPER contributes to VEGF regulation in cancer cells exposed to copper [164]. Further extending the cooperation between GPER and HIF-1α in tumor progression and invasion, we reported that in normoxic breast cancer cells estrogen-activated GPER induces HIF-1α expression and its target gene VEGF [165]. Furthermore GPER also mediated the effect of endothelin-1 (ET-1) on HIF-1 expression in normoxic ER negative breast cancer cells [166]. As discussed in the next section, the mechanism through which GPER signaling induces HIF-1α expression remains to be elucidated, however our studies demonstrated that the cross-talk between GPER and the Epidermal Growth Factor Receptor (EGFR) signaling plays a relevant role in inducing HIF-1α protein levels [165]. In this regard, in ER-negative breast cancer cells and CAFs, estrogenic GPER signaling was shown to activate EGFR/ERK transduction cascade, leading to the increase of c-fos [165], which is known to regulate gene transcription by binding to the AP1 consensus sequence in the promoter of target genes, together with members belonging to the c-jun family [167]. The GPER triggered EGFR/ERK/c-fos transduction pathway was shown to mediate the transcriptional up-regulation of HIF-1α by estrogen, possibly activating the AP-1 sites within the HIF-1α promoter [165]. Likewise, breast cancer cells engineered to overexpress a mutant c-fos, unable to bind the AP-1 sites, failed to induce the up-regulation of HIF-1α in response to estrogen [165], thus supporting the involvement of the transcription factor c-fos in the transcriptional activation of HIF-1α gene.

## 10. HIF-1α Regulation by E2 (ER e GPER)

The bi-directional and reciprocal interplay between estrogen and hypoxia signaling has been shown to regulate a number of physio-pathological functions in cellular and animal models. For instance, in rat uterus both ERα and HIF-1α were found to be recruited to the HRE consensus sequences located within the VEGF promoter upon stimulation with E2 during normoxia [168]. In addition, ERα mediates estrogenic regulation of HIF-1α in ovarian and breast cancer cells, breast cancer associated fibroblasts and in the rat uterus [165,169,170,171]. On the other hand, HIFs appear to regulate the expression and functionality of ERs, including the classic ERα and ERβ, as well as the alternate estrogen receptor GPER [172,173,174]. Corroborating the hypothesis of a complex molecular interdependence between ER- and HIF-mediated pathways, an increased expression of HIF-1α has been correlated with a more aggressive phenotype in ER positive breast cancer [175]; furthermore HIF-1β serves as a potent coactivator of ER-mediated gene transcription [176]. Recently, a comprehensive overview of HIF-1α action on ERα expression and function was provided in a selected panel of ER-positive breast cancer cells, which were representative of the diverse genetic backgrounds and mutational landscape commonly observed in ER-α positive tumors. Results from this investigation show that low environmental O_2_ triggers HIF-1α activity toward a rapid reduction of ERα protein levels, due to enhanced proteolysis [172]. These data add to previous studies showing that in breast cancer cells as well as in human breast tumor samples HIF-1α lowers ERα levels by repressing its transcription upon stimulation with hypoxia or hypoxia-mimetic agents [177,178,179]. Altogether these observations have profound clinical implications, as pharmacological strategies aimed at inhibiting HIF-1α action and/or proteasomal degradation might represent useful strategies for stabilizing ERα expression and sensitize certain sub-populations of breast cancer patients to endocrine therapies [180].

Extending the framework of these findings, estrogens were reported to regulate HIF-1α activity both in normoxic and hypoxic conditions in breast cancer. For instance, in normoxic conditions E2 led to the recruitment of ERα to VEGF promoter, hence inducing VEGF expression [181]. Differently, in hypoxic conditions VGEF expression was driven by ER-dependent recruitment of HIF-1α to its promoter [181]. Of note, in human breast cancer cells, a rapid estrogen action involving ERα-mediated activation of the c-Src/PI3K/AKT/mTOR pathway was accountable for the up-regulation of HIF-1α protein expression [182].

On the other hand, HIFs-dependent effects can be differentially regulated in ERα-positive compared to ERα-negative breast tumors [183]. For instance, in ERα-positive tumors hypoxia increased CSCs activity in an ERα/Notch-dependent fashion, whereas it decreased CSCs in ER-α negative context [183]. Furthermore, it has been elucidated that many of the HIF-1α target genes bear both hypoxia- and estrogen-response elements, and are regulated by ERα in both normoxic and hypoxic conditions [184]. Completing this intricate puzzle, a functional ERE sequence was detected also within the HIF-1α promoter [184]. Of note, in ERα breast tumor patients, a hypoxia metagene signature together with upregulated HIF-1α expression were associated with lower response to endocrine treatment, corroborating the importance of ERα/HIF-1α crosstalk in the hormone response to treatment [185].

Despite the stimulatory role mediated by E2/ERα signaling on HIF-1α expression in breast cancer, the interaction between estrogen signaling and HIF-2α shows an opposite trend. Indeed, E2 causes down-regulation of both HIF-2α mRNA and protein expression in ER-positive breast cancer cells, but not in ER-negative cells. This negative regulation possibly involves an ERα-mediated recruitment of co-repressors to the estrogen response element (ERE) of the HIF-2α promoter [186].

It should be mentioned that ERβ has been associated with reduced HIF-1 transcriptional activity during hypoxia in the diverse cancer cell context, including breast cancer [187,188]. Nevertheless certain discrepancies on the role of ERβ in modulating HIF-1 activity are reported in the literature; for instance, ERβ was positively correlated with HIF-1α in primary and metastatic breast tumors [189]. Furthermore, the human ERβ variant ERβ2 was negatively associated with PHD3, but positively associated with HIF-1α, toward aggressive features in triple negative breast cancer cells [190].

As discussed earlier in this review, beyond ERα and ERβ, estrogens signal through the G-protein coupled estrogen receptor, a member belonging to the rhodopsine-like family of G-protein coupled receptors (GPCRs) [70]. As mentioned above, hypoxia is associated with both aggressive features of breast cancer and down-regulation of ERα pathway, suggesting that in low oxygen microenvironment estrogens might signal through GPER to overcome ERα loss. If so, GPER expression should be induced by hypoxia. Indeed, a bioinformatic analysis of the human GPER promoter has revealed HRE consensus sequences, suggesting that GPER might be regulated in hypoxic conditions in a HIF1α-dependent manner. This hypothesis was then verified in ER-negative breast cancer cells, where the exposure to low oxygen tension (2% O_2_), as well as the treatment with the hypoxia mimetic CoCl_2_ triggered HIF-1α accumulation and its recruitment to the GPER promoter, together with the subsequent up-regulation of GPER protein expression [174]. Interestingly, a functional cooperation between HIF-1α and GPER in breast cancer cells and in breast cancer associated fibroblasts was shown to regulate the hypoxia-dependent VEGF expression toward tumor angiogenesis and progression [191]. Furthermore, hypoxia stimulates CAFs to secrete paracrine factors including IL-6, VEGF and CTGF, in a HIF-1α/GPER dependent manner [192]. These observations suggest that GPER, similarly to ERα, may serve as a further regulator of the hypoxic pathway through a functional crosstalk with HIF-1α.

As mentioned in the previous section, we found that estrogenic GPER signaling activated the HIF-1α/VEGF transduction pathway in normoxic ER-negative breast cancer cells and CAFs, through the engagement of the EGFR/ERK/c-fos cascade [165]. This engagement of GPER was required for boosting new blood vessel formation and lead tumor growth in a mouse xenograft model of breast cancer [165].

These observations extend previous findings showing that hormones and growth factors may regulate HIF-1-dependent functions in normoxia through several mechanisms that include HIF-1α gene transcription, protein translation, as well as protein stabilization [160,161]. Additional investigations should clarify whether the use of proteasome inhibitors may have a deeper impact on GPER-mediated HIF-1α functions in normoxic conditions, evidencing whether HIF-1 protein stabilization rather than gene transcription might play a more relevant role in GPER action. Recently, Yu and collaborators demonstrated that GPER signaling triggers aerobic glycolysis in breast CAFs, leading to the accumulation of the end by-products pyruvate and lactate [193]. As these fuels may act as oncometabolites in cancer cells, it is conceivable to hypothesize that the O_2_-independent regulation of HIF-1α by GPER may result from the activation of the glycolytic pathway and the subsequent production of pyruvate and lactate, which are known to stabilize HIF-1α in normoxia [194,195,196], generating a condition known as pseudo-hypoxia [158].

Evidence for a stimulatory role exerted by E2-activated GPER on HIF-1α expression was obtained also in certain physio-pathological conditions associated with de-regulated estrogen signaling as endometriosis [197]. Recently, Xu et al. demonstrated that in a hypoxic microenvironment, BPA promotes proliferative effects in both breast cancer cell and endothelial cells by inducing HIF-1α and VEGF expressions in a GPER-mediated manner [198]. These data suggest that a molecular loop connecting HIF-1α and GPER supports breast cancer progression in a multilayered fashion that includes components of both cancer and stromal cells. On the other hand, a tumor-suppressive role for the HIF-1α/GPER signaling was demonstrated in certain sub-populations of breast cancer recapitulating the molecular features of the triple negative subtype. In this regard, in vitro and in vivo evidence showed that GPER blocks cell cycle progression, induces apoptosis, inhibits migration and invasion, and reverses the process of EMT in the context of TNBC (Triple Negative Breast Cancer) [199,200]. In addition, the selective activation of GPER by its synthetic ligand G-1 mediates anti-angiogenic effects by repressing the HIF-1α/VEGF signaling in the triple negative MDA-MB-231 breast cancer cells [201], thus suggesting that the tumor promoting/tumor suppressive role of the HIF-1α/GPER signaling in breast cancer may at least in part depend on the ER and HER2 status.

The translational implications of the interdependence between estrogenic and hypoxic signaling mediated by ER and/or GPER together with HIF-1α could deeply impact the development of novel combination strategies aimed at inhibiting breast cancer progression and overcoming anti-cancer drug resistance. Further studies are required to better clarify the involvement of ERs and HIF-1α cross-talk in the regulation of key events involved in breast metastasis and therapeutic escape. For instance, it has been reported that HIFs are involved in the establishment of both chemo- and endocrine-resistance [163,202] by facilitating the enrichment of breast cancer stem cells populations, regardless of ER status.

## 11. Does a Signaling Network between Notch, HIF-1α, and GPER Strengthen EMT in Breast Cancer Cells?

**Figure 2:**
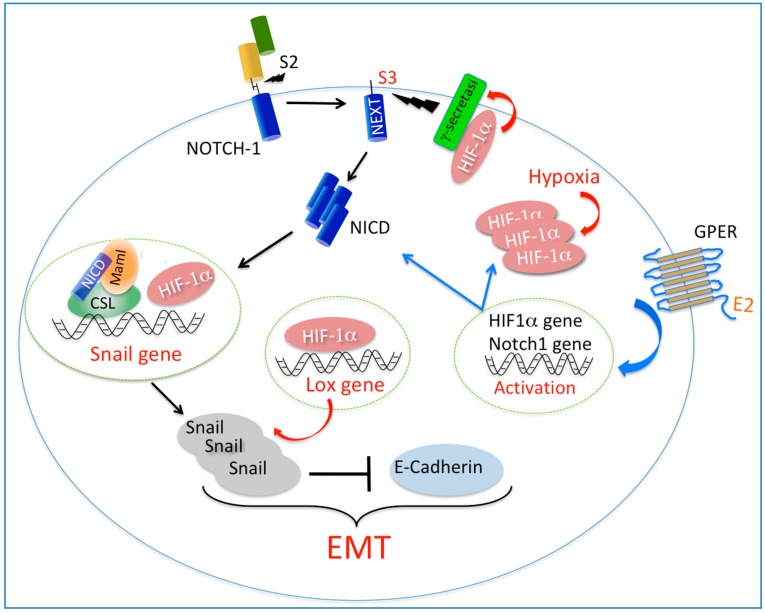
Proposed model for a signaling network between Notch, HIF-1α, and GPER in cancer epithelial-mesenchymal-transition (EMT). In hypoxic breast cancer cells, HIF-1α accumulation increases γ-secretase activity and in turn NICD generation. In the nucleus, NICD and HIF-1α cooperates for the induction of Snail gene expression. Also, HIF-1α indirectly regulates Snail protein stability by inducing the expression of *LOX* gene. E2-activated GPER may potentiate the effect of hypoxia on Notch-dependent expression of Snail by directly inducing transcriptional activation of both Notch-1 and HIF-1α. In normoxic breast cancer cells, E2/GPER induces both Notch-1 expression and activation of the Notch core pathway, hence leading to the induction of Snail-1 expression. In addition, E2/GPER may potentiate Notch-dependent expression of Snail by inducing HIF-1α expression and HIF-1α-dependent transcriptional activity. Red arrows indicate mechanisms shown in hypoxic cells; blue arrows indicate mechanisms shown in normoxic cells; black arrows indicate mechanisms shown in both normoxic and hypoxic cells.

Besides conferring migratory and invasive features to epithelial cancer cells, the induction of cancer EMT is also linked to the gaining of other properties, including enhanced stem cell and chemotherapy resistance. The extent of these acquired cellular capabilities by cancer cells is dependent on the type and strength of the signal inducing EMT. In cancer EMT, signaling networks are of particular importance for the generation of robust cellular responses to microenvironmental variations. For example, the functional interplay of Notch signaling with hypoxia and TGFβ has been suggested to induce a more rapid and pronounced EMT in cancerous epithelial cells [52,203]. Individually, hypoxia and TGFβ signaling pathways have been shown to induce Notch-mediated EMT by different mechanisms that converge at the level of NICD [204]. Likely, if such signaling pathways are simultaneously active in the cell, they may result in a more robust EMT [203,204]. In breast cancer cells, the effect of hypoxia on Notch-mediated EMT is mediated by HIF-1α recruitment at the Snail promoter, which in turn potentiates NICD/CSL/MAML-dependent expression of the Snail gene [38] (Figure 2). Similarly, estrogen/GPER signaling leads to the induction of Snail expression in a NICD/CSL/MAML-dependent manner [6], suggesting that GPER may cooperate with HIF-1α to potentiate Notch-dependent expression of Snail in breast cancer cells exposed to both hypoxia and estrogen. Indeed, GPER was shown to cooperate with HIF-1α for the expression of invasive markers, including CTGF, VEGF and IL-6 in both breast cancer cells and CAFs exposed to low oxygen [191,192]. Interestingly, in CAFs derived from breast carcinomas, estrogen induces GPER translocation to the nucleus and its recruitment at promoters of genes involved in cell migration and angiogenesis [78,174,191,205]. In particular, it was shown that nuclear GPER potentiates HIF-1α recruitment at the VEGF promoter, in turn increasing VEGF expression and activity [191]. Collectively, these observations suggest that estrogen could possibly strengthen Notch-mediated EMT by increasing HIF-1α recruitment at the Snail promoter via nuclear GPER. On the other hand, studies in ER-negative breast cancer SkBr3 cells showed that estrogen/GPER signaling is able to induce HIF-1α expression and activity [165] as well as Notch-1 mRNA levels and NICD accumulation [6], suggesting that estrogen/GPER may strengthen EMT by simultaneously inducing Notch and HIF-1α signaling pathways in normoxic breast cancer cells (Figure 2). A further understanding of how hypoxia and estrogen converge on Notch signaling activation will provide further information on how intracellular signal networks mediate information from the cellular microenvironment to the cell nucleus to support a robust EMT in breast cancer cells.

## 12. Therapeutic Approaches Targeting Notch Signaling in Breast Cancer

The evidence that deregulation of the Notch signaling pathway favorites tumor growth and invasiveness has led to the development of various therapeutic approaches targeting different molecules along this pathway [3,206]. These include a variety of γ-secretase inhibitors (GSI) [206], antibodies against Notch1-3 receptors [207,208] or DLL4 ligand [209], Notch soluble decoys targeting Notch1, and DLL1 [210,211]. Other types of experimental inhibitors have been developed to inhibit the NICD transcriptional activation complex and consist of synthetic peptides or small inhibitor molecules blocking the interaction of MAML-1 with the NICD/CSL transcriptional complex [212,213]. Also, further small molecules targeting the Notch endocytic pathway have been screened and shown to inhibit endogenous Notch signaling in vitro and in vivo [214], or to selectively target the endocytic maturation of oncogenic Notch-1 mutations [215].

The interplay of the Notch signaling with several others context-specific tumorigenic pathways has prompted the usage of GSIs in combination therapies for different types of solid tumors [206]. Earlier studies in ERα-positive breast cancer cells indicated that the combination of antiestrogen with GSI might be a potential therapeutic strategy in clinical studies [61]. Similarly, studies in breast tumor xenografts generated by ErbB-2 positive BTA474 breast cancer cells suggested that the combination of trastuzumab with GSI could decrease the recurrence of ErbB-2-positive breast tumors and may be beneficial in the treatment of recurrent trastuzumab-resistant disease [216]. Furthermore, both preclinical and clinical studies showed the combination of GSI with docetaxel targets breast cancer stem cells in human breast tumors [217]. Similarly, the combination of the reversal GSI inhibitor PF-03084014 with docetaxel inhibits tumor growth in patient-derived xenograft models of triple-negative breast cancer [218]. GSI also inhibits the tumorigenic effect of hypoxia in breast cancer progression and invasiveness [98], suggesting that it can potentially be used in combination with chemotherapy to prevent or delay the onset of metastatic breast malignancy.

In perspective, further identifications of context-specific Notch-interacting partners could be crucial for the development of more targeted antitumor therapy protocols with fewer side effects. In this regard, more insights into the signaling network between Notch, HIF-1α, and GPER in breast cancer EMT could provide new hints for the generation of novel combination strategies targeting breast cancer progression and overcoming anti-cancer drug resistance.
